# Study on the psychological health status and influencing factors of operating room nursing staff

**DOI:** 10.1097/MD.0000000000038780

**Published:** 2024-07-19

**Authors:** Lan Yao, Linjuan Zhang

**Affiliations:** a Department of Anesthesia Surgery, The First Affiliated Hospital of Xi’an Jiaotong University, Xi’an, Shaanxi, China.

**Keywords:** influencing factors, operating room nurses, physical health satisfaction, psychological health, subhealth symptoms

## Abstract

To comprehensively analyze the psychological health status of operating room nurses and identify influencing factors. The research compares psychological health differences based on nurses’ years of experience, specifically examining depression and anxiety scores. A detailed assessment was conducted, focusing on nurses with varying experience levels. Findings revealed higher depression scores among mid to senior-level nurses, while junior-level nurses exhibited elevated anxiety scores. Additionally, overall dissatisfaction with physical health and various subhealth symptoms were reported. Multifactorial analysis identified working hours, disaster relief experience, and perceived occupational benefits as primary influencers. Through comparative analysis, it was found that the average score of self-rating depression scale and self-rating anxiety scale was 53.8 ± 12.2 points and 47.6 ± 10.5 points respectively. The depression score of middle and senior nurses was significantly higher than that of junior nurses (*P* < .05). The anxiety score of primary nurses was significantly higher than that of middle and senior nurses (*P* < .05). The results indicate that the duration of work, previous experience in disaster relief, and nurses’ perception of occupational benefits were the main factors influencing the psychological health status of operating room nurses (*P* < .05). Healthcare institutions are recommended to implement targeted interventions based on nurses’ experience levels, addressing specific psychological health needs. Future research should delve into specific subgroups, conduct long-term tracking, expand the scope of influencing factors, assess the effectiveness of intervention measures, and explore cross-cultural aspects.

## 1. Introduction

The operating room is a highly specialized and demanding workplace within a hospital, characterized by complex medical tasks, technical precision, and a fast-paced environment.^[1,2]^ Operating room nursing staff, as crucial assistants to doctors and guardians of patient safety, bear a substantial psychological burden surpassing that of general medical positions.^[3]^ The challenges they face stem from the need for solid medical knowledge, excellent nursing skills, adaptability, and keen observational abilities during surgeries.^[4,5]^

Work pressure in the operating room is significant, as the success of a surgery directly impacts patient safety and the hospital’s reputation.^[6,7]^ Nursing staff must maintain constant vigilance, devoid of negligence or complacency, while also navigating the complex emotions of patients and their families. The intense nature of the work further takes a toll on their physical and mental well-being, leading to fatigue, anxiety, and potential mental health issues.^[8]^ Additionally, the unique demands of the operating room often force nursing staff into continuous overtime and on-call duties, perpetuating a constant state of tension.

Recognizing the importance of psychological health for nursing professionals,^[9,10]^ this study aims to comprehensively understand the psychological well-being of operating room nursing staff and the influencing factors. The focus includes an examination of work stress, burnout, anxiety, depression, quality of life, sleep conditions, and overall well-being through methods such as questionnaire surveys and psychological tests.^[9,11–14]^

In-depth analysis will explore how various factors, including the work environment, job stress, career development, and personal qualities, impact the psychological health of nursing staff. This encompasses issues such as the sufficiency of rest spaces in the operating room, manageability of work stress, availability of career development opportunities, and potential challenges related to personal qualities such as emotional management skills and character flaws.

The study aims to provide a scientific foundation for improving the working conditions, job satisfaction, and patient safety levels for operating room nursing staff. Recommendations will be formulated based on a profound understanding of psychological health status and influencing factors. These may include optimizing the work environment, scheduling tasks to alleviate stress, establishing comprehensive career development pathways, and reinforcing psychological health education and training. In summary, the research aspires to draw attention and concern from hospital management and the broader community, collectively contributing to the enhancement of operating room nursing quality and ensuring patient safety.^[9,11–14]^

## 2. Research design and methods

This study was approved by the Ethics Committee of the First Affiliated Hospital of Xi’an Jiaotong University. This survey focuses on operating room nursing staff with the aim of comprehensively assessing their psychological health status and related influencing factors. The survey encompasses nursing staff from different hospitals, positions, work experiences, genders, and age groups to ensure the breadth and representativeness of the data.

To obtain accurate and comprehensive data, we meticulously designed various survey tools. Firstly, a self-designed questionnaire covering personal information, sources of work stress, feelings of occupational burnout, and quality of life assessment, among other dimensions, was created to delve into the psychological health status and influencing factors of nursing staff. Secondly, internationally recognized standardized psychological scales such as the self-rating anxiety scale and the self-rating depression scale were employed to objectively assess the psychological health levels of nursing staff.^[15–17]^ Additionally, to understand the actual working environment in the operating room and the daily work status of nursing staff, a detailed observation record sheet was developed to document work processes, interactions, and emotional changes.

In terms of research methods, a combination of various approaches was employed. Firstly, self-designed questionnaires and standardized psychological scales were distributed to participants in online or paper form, requesting them to fill in according to their actual situations. To ensure the authenticity and completeness of the data, emphasis was placed on anonymity and confidentiality to alleviate concerns among nursing staff. Secondly, standardized psychological scales were used for psychological assessments, with professional psychologists responsible for analyzing and interpreting the assessment results. Furthermore, on-site observations were conducted to record work processes, interactions, and emotional changes of nursing staff, providing firsthand information. Lastly, representative nursing staff was selected for in-depth interviews to gain insights into their work experiences, psychological health issues, and coping mechanisms through face-to-face communication.

In terms of data analysis, a series of statistical methods were employed. Firstly, descriptive statistics were used to calculate mean values, standard deviations, frequencies, and other parameters to describe the basic characteristics and psychological health status of the surveyed individuals. Secondly, *t* tests and analysis of variance were conducted to compare the psychological health differences among nursing staff in different groups. Additionally, correlation analysis was performed to explore the relationships between factors such as work stress, occupational burnout, and quality of life with psychological health status. Regression analysis was employed to identify key factors influencing the psychological health of operating room nursing staff and establish predictive models. Finally, path analysis revealed direct and indirect pathways between various influencing factors and their comprehensive impact on psychological health status.

## 3. Results

### 3.1. Psychological health status of nurses with different seniority

In this survey, we paid particular attention to the psychological health status of nurses with different levels of seniority. Through comparative analysis, it was found that the average score of the self-rating depression scale for operating room nurses was 53.8 ± 12.2, and the average score of the self-rating anxiety scale was 47.6 ± 10.5 (Table 1). Notably, the depression scores of mid to senior-level nurses were significantly higher than those of junior-level nurses, showing statistically significant differences. Conversely, the anxiety scores of junior-level nurses were significantly higher than those of mid to senior-level nurses, also exhibiting statistically significant differences.

**Table 1:** SDS and SAS evaluation results of different years.

Age	n	SDS		SAS	
		Score	Positive rate (%)	Score	Positive rate (%)
1–5 yr	58	45.9 ± 11.3	12(21)	55.6 ± 10.1	50(86)
6–15 yr	66	58.2 ± 10.7	50(76)	48.9 ± 12.6	40(60)
>16 yr	10	62.1 ± 13.6	8(80)	42.1 ± 11.4	2(20)
*t*		4.52		5.15	
*P*		<.01		<.01	

SAS = self-rating anxiety scale, SDS = self-rating depression scale.

### 3.2. Health satisfaction status

In this survey, we observed a relatively low level of physical health satisfaction among operating room nurses, with only 32.7%. This result indicates that the physical health status of operating room nurses requires more attention and emphasis. Additionally, various subhealth symptoms were identified among nurses, further highlighting the severity of their physical health issues. Particularly noteworthy is the highest proportion of positive responses to self-reported discomfort symptoms among mid-level nurses (Table 2). This finding may be related to the greater work and life pressures faced by mid-level nurses, as they are often at a critical juncture in their careers. They not only bear more work responsibilities but may also contend with multiple pressures in family life, such as child education and parental care. These factors may contribute to a higher likelihood of physical discomfort and subhealth symptoms.

**Table 2:** The positive rate of physical discomfort in different years (n, %).

Age	n	Gastritis and gastric discomfort	Cervical spondylosis	Lumbar disc herniation	Neurasthenia	Obsessive -compulsive disorder
1–5 yr	58	10(17)	0	0	16(28)	38(66)
6–15 yr	66	38(58)	4(6)	2(3)	54(82)	60(91)
>16 yr	10	6(60)	0	0	6(60)	8(80)
*χ^2^*				37.11		
*P*				<.01		

### 3.3. Multifactorial analysis of factors influencing nurses’ psychological health

Using psychological health status (0 representing negative and 1 representing positive) as the dependent variable, logistic backward-LR regression analysis was conducted (Table 3). The results indicate that the duration of work, previous experience in disaster relief, and nurses’ perception of occupational benefits were the main factors influencing the psychological health status of operating room nurses (*P* < .05), as shown in Table 4. In the regression equation, these 3 factors significantly impacted the psychological health status.

**Table 3:** Assignment table.

Factor	Assignment
Cause variable	Negative = 0, positive = 1
Psychological assistance needs	Need = 1, no = 2, no consideration = 3
Experience similar to disaster rescue	Yes = 1, no = 2
Children’s situation	There is = 1, no = 2
Length of work	≤6 h = 1, ≥8 h = 2
Job title	Primary titles = 1, intermediate and above job title = 2
Nurse career benefits	Measured value

**Table 4:** Multifactors affecting the mental health of the operating room.

Variable	B	SE	Wald	*P*	OR	95% CI
Experience similar to disaster rescue	0.939	0.441	4.522	.033	0.391	0.165, 0.929
Length of work	0.751	0.331	5.144	.023	0.472	0.247, 0.903
Nurse career benefits: score	−0.069	0.019	13.581	0	0.934	0.900, 0.968
Constant	4.287	1.157	13.724	0	72.776	

## 4. Discussion

### 4.1. Analysis of psychological health status among nurses with different seniority

As one of the core departments in a hospital, the working environment and nature of work in the operating room significantly impact the psychological health of nurses. In this survey, we found significant differences in the psychological health of operating room nurses with varying levels of seniority, providing valuable data for an in-depth exploration of nurses’ psychological health issues.^[18–20]^

Firstly, mid to senior-level nurses showed significantly higher depression scores than junior-level nurses. This could be attributed to the greater pressures faced by mid to senior-level nurses. They not only bear more work tasks and responsibilities but also grapple with more complex career development issues and higher family responsibilities. Additionally, with increasing age, they may encounter more health-related problems, which could adversely affect their psychological well-being. Therefore, healthcare institutions should pay special attention to the psychological health of mid to senior-level nurses, providing them with additional psychological support and intervention measures, such as regular psychological assessments, counseling services, and career planning guidance, to help them better cope with the pressures and challenges in work and life. Secondly, junior-level nurses exhibited higher anxiety scores. This may be associated with their recent entry into the workforce and not yet fully adapting to the work environment and role expectations. As newly employed nurses, they may need more time and effort to familiarize themselves with work processes, acquire professional skills, and establish positive interpersonal relationships. In this process, they may experience anxiety, unease, and high levels of stress. Therefore, healthcare institutions should enhance training and support for junior-level nurses, assisting them in quickly adapting to the work environment and role expectations, thereby boosting their confidence and job satisfaction. Additionally, initiatives like mentorship programs and providing career development opportunities can stimulate the enthusiasm and motivation of junior-level nurses. In addition to implementing tailored psychological health support and intervention measures for nurses at different seniority levels, healthcare institutions should strengthen overall psychological health management and educational efforts. This includes conducting regular psychological health lectures and training activities to raise nurses’ awareness and importance of psychological health issues, establishing a robust psychological assessment and intervention mechanism to promptly identify and address nurses’ psychological health problems, and fostering a positive work atmosphere and team culture to enhance nurses’ sense of belonging and cohesion.

### 4.2. Health satisfaction and subhealth symptoms

In this survey, the health satisfaction of operating room nurses was only 32.7%, a concerning result that reflects nurses’ dissatisfaction and concerns about their own physical health. As crucial members of the medical team, operating room nurses face high-intensity work pressures and a stressful working environment, factors that may adversely affect their physical health. Additionally, we identified various subhealth symptoms among nurses. Subhealth status refers to a condition where the body is in a state between health and illness, characterized by feelings of physical discomfort, mental fatigue, and easy fatigue, despite the absence of obvious disease manifestations. While these symptoms may not directly impact nurses’ work capabilities, they pose a potential threat to their overall physical and mental well-being. Particularly, mid-level nurses had the highest positivity rate in self-reported discomfort symptoms, possibly linked to their greater work and life pressures, and more prominent issues in balancing family and work responsibilities.

To enhance nurses’ health satisfaction and reduce the occurrence of subhealth symptoms, healthcare institutions should implement a series of measures.^[21–24]^ Firstly, there should be an emphasis on health education and awareness campaigns for nurses to raise their understanding and importance of health issues. Secondly, optimizing the work environment and workflow to minimize unnecessary work pressure and fatigue is crucial. This can include appropriately scheduling work and rest periods and providing comfortable workspaces and equipment. Additionally, necessary health support and resources, such as regular health checkups and health counseling services, should be provided to help nurses promptly identify and address physical health issues. In addition to institutional support and management, nurses should actively focus on and maintain their own physical health. This can involve maintaining good sleep and dietary habits, engaging in appropriate physical exercise and relaxation activities, and fostering a positive mindset and emotions. Mutual care and support among nurses can also contribute to collectively addressing workplace stress and challenges. In summary, improving the health satisfaction of operating room nurses and reducing the occurrence of subhealth symptoms are crucial tasks in healthcare institution management. Only through strengthening health education, optimizing the work environment, and providing health support measures can better protection and support be offered for the physical and mental health and professional development of nurses. Additionally, continuous exploration and innovation in health management and support methods are needed to provide more comprehensive and effective protection for nurses’ physical health.

### 4.3. Multifactorial analysis of factors influencing nurses’ mental health

While exploring the mental health of operating room nurses, understanding the impact of different seniority levels or specific factors is insufficient. To gain a more comprehensive understanding, we conducted a multifactorial analysis to identify additional potential influencing factors and provide more targeted recommendations for improving nurses’ mental health. Through logistic regression analysis, we identified the main factors influencing the mental health of operating room nurses as work duration, experiences similar to disaster relief, and nurses’ perceived professional benefits.^[25–27]^

Firstly, work duration showed a negative correlation with nurses’ mental health status. Prolonged working hours not only lead to physical fatigue but may also trigger emotional and psychological stress. Therefore, optimizing work processes, enhancing efficiency, and appropriately scheduling work and rest times are crucial for improving nurses’ mental health. Secondly, experiences similar to disaster relief also impact nurses’ mental health. Nurses involved in disaster relief efforts may face additional psychological trauma and stress, requiring more psychological support and intervention. Healthcare institutions should establish corresponding psychological assistance mechanisms to provide timely counseling and support for these nurses. Additionally, the perceived professional benefits score was identified as a significant influencing factor. A lower score in professional benefits may lead nurses to feel uncertain and anxious about their career prospects and development, subsequently affecting their mental health. Hence, healthcare institutions should strengthen nurses’ career planning and training, offer more professional development opportunities and resources, assist nurses in clarifying their career direction, and enhance professional satisfaction and a sense of achievement. While these 3 factors were highlighted, there may be other potential influencing factors, such as individual personality, family situation, and work environment. To gain a more comprehensive understanding of how these factors impact nurses’ mental health, further in-depth research and analysis are necessary.

### 4.4. Recommendations for improvement and future prospects

Based on the analysis results, we propose the following recommendations.

1. Establish a comprehensive mental health support system: Healthcare institutions should establish a robust mental health support system, including regular psychological assessments and interventions, and providing psychological counseling services. This can help nurses identify and address psychological issues promptly, ultimately improving their mental health.
2. Optimize the work environment and processes: By optimizing the work environment and processes, reducing unnecessary work stress and overtime, healthcare institutions can create a more comfortable and efficient working environment for nurses. Additionally, scheduling work and rest times reasonably ensures that nurses have sufficient time for rest and relaxation.
3. Strengthen career planning and training: Healthcare institutions should enhance career planning and training programs for nurses, offering more professional development opportunities and resources. This can assist nurses in clarifying their career goals, enhancing professional satisfaction, and promoting a sense of achievement, ultimately contributing to improved mental health.
4. Establish a psychological aid mechanism: Specifically for nurses with unique experiences such as participating in disaster relief, healthcare institutions should establish a corresponding psychological aid mechanism. This mechanism would provide timely psychological counseling and support to help nurses cope with the emotional challenges associated with such experiences. These recommendations aim to create a supportive and conducive environment for nurses, acknowledging and addressing the various factors influencing their mental health. Looking forward, continuous research and innovative approaches in health management and support should be explored to provide holistic and effective protection for nurses’ mental and physical well-being.

Recommendations for Future Research:

1. In-depth study of specific nurse groups: Future research can focus on specific groups of nurses (such as newly hired nurses, night shift nurses, ICU nurses, etc) to more precisely identify their needs and issues.
2. Longitudinal tracking studies: Conducting long-term tracking studies on nurses to understand trends in their psychological health over time and in response to changes in the work environment will provide a basis for more effective interventions.
3. Expanding the scope of influencing factors: In addition to known factors, future research can explore other potential factors, such as individual personality, family situations, and work environment, to gain a more comprehensive understanding of the influencing factors on nurses’ psychological health.
4. Effectiveness evaluation of intervention measures: Assessing the actual effects of proposed recommendations and intervention measures will help determine which interventions are most effective, allowing for continuous improvement and optimization.
5. Cross-cultural studies: Considering cultural differences across countries and regions, future research can conduct cross-cultural studies to understand variations and commonalities in the psychological health of nurses in different cultural contexts.

## 5. Conclusion

In summary, our study revealed noteworthy distinctions in the psychological health of operating room nurses based on their experience levels. Nurses with intermediate to high experience exhibited higher depression scores, while less experienced nurses reported elevated anxiety. Paying attention to the psychological health of nurses and implementing corresponding support and intervention measures is crucial for enhancing medical quality and nurses’ job satisfaction. Future research should continue to delve deeper into this field to provide stronger support for practical applications.

## Acknowledgments

We would like to thank the participants of this study for sharing their experiences.

## Author contributions

**Conceptualization:** Lan Yao, Linjuan Zhang.

**Data curation:** Lan Yao, Linjuan Zhang.

**Formal analysis:** Lan Yao.

**Investigation:** Lan Yao, Linjuan Zhang.

**Methodology:** Lan Yao, Linjuan Zhang.

**Supervision:** Linjuan Zhang.

**Writing** – **original draft:** Lan Yao, Linjuan Zhang.

**Writing** – **review & editing:** Lan Yao, Linjuan Zhang.
